# Change in Concentration of Amorphous Region Due to Crystallization in PTT/PET Miscible Blends

**DOI:** 10.3390/polym16162332

**Published:** 2024-08-17

**Authors:** Kousuke Sugeno, Hiromu Saito

**Affiliations:** Department of Organic and Polymer Materials Chemistry, Tokyo University of Agriculture and Technology, Koganei-shi, Tokyo 184-8588, Japan; s228409z@st.go.tuat.ac.jp

**Keywords:** PTT, PET, blend, crystallization, concentration, amorphous region

## Abstract

In a miscible crystalline/crystalline blend of poly(trimethylene terephthalate) (PTT) and poly(ethylene terephthalate) (PET), the PET spherulites grew at 240 °C when the PTT content was 30 wt% or less. The growth rate of PET spherulites decreased with time due to the exclusion of PTT from the growth front of PET spherulites into the amorphous region, resulting in a three-stage crystallization process. Due to the exclusion, the spherulite growth stopped before the volume filling of the PET spherulites, causing the formation of an excluded PTT amorphous region. When the temperature was lowered from 240 °C to 210 °C, the PTT spherulites grew in the excluded PTT amorphous region. The spherulite growth rate of PTT in the excluded PTT amorphous region was equivalent to that of a blend of 60–70 wt% PTT in 30/70 PTT/PET. These results suggest a significant change in the PTT concentration in the amorphous region, from the initial PTT content of 30 wt% to 60–70 wt%, due to the exclusion of PTT during the melt crystallization of PET at 240 °C.

## 1. Introduction

In polymer blends, two or more different polymers are mixed to create new materials. Many of the polymer blends used in applications contain crystallizable polymers. If the component polymers are miscible and one of the components is crystallizable while another is not, the non-crystallizable components will diffuse away from the crystal growth front of the crystallizable component into the amorphous region during the crystallization of the crystallizable component. This phenomenon is called exclusion from the crystal growth front, and it has been observed in numerous studies [1,2,3,4,5,6,7,8,9,10,11,12,13,14,15,16,17,18,19]. When the exclusion is confined to the interlamellar amorphous region, the non-crystallizable component is inserted into the interlamellar amorphous region [20,21,22,23,24]. When the degree of exclusion is increased either by increasing the content of the non-crystallizable component or by increasing the crystallization temperature, the non-crystallizable component is excluded from the interlamellar amorphous region and diffused into the interfibrillar or interspherulite amorphous region [25,26,27]. When the non-crystallizable component is confined to the interfibrillar amorphous region, the spherulite morphology becomes coarse and open [9,14,15,28,29,30,31,32,33,34]. Conversely, when the non-crystallizable component diffuses into the interspherulite amorphous region and is not confined to the interfibrillar amorphous region, no significant change in spherulite morphology is observed [8,35].

As the non-crystallizable component diffuses from the interlamellar amorphous region into the interfibrillar or interspherulite amorphous region, the concentration of the excluded non-crystallizable component at the spherulite growth front increases during the spherulite growth process. The increased concentration of the excluded non-crystallizable component causes the spherulite growth rate to decrease. In other words, the spherulite grows nonlinearly with time. This is in contrast to the linear growth commonly observed in neat crystalline polymers, where the spherulite grows linearly with time. Nonlinear spherulite growth has been observed in a number of different polymer blends, including poly(butylene terephthalate)/poly(e-caprolactone) [12], poly(butylene succinate)/poly(butylene adipate) [4,7,14], poly(vinylidene fluoride) (PVDF)/poly(methyl methacrylate) (PMMA) [9,15], and polypropylene (PP)/liquid paraffin (LP) blends [8]. Spherulite growth stops before the volume filling of the spherulites when the non-crystallizable component is excluded into the interspherulite amorphous region and the amorphous melt region is enriched with a substantial amount of the excluded non-crystallizable component [8,32,36]. In contrast, if the non-crystallizable component is retained in the interlamellar or interfibrillar amorphous region, as observed in PVDF/PMMA blends [9,15], the spherulite growth typically continues until the spherulites are volume-filled. Accordingly, the concentration of the excluded non-crystallizable component in the amorphous melt region is considered a critical factor in understanding the exclusion behavior during the crystallization of polymer blends. However, despite theoretical discussions on the concentration of the excluded non-crystallizable component [1,6,12,26], the concentration of the excluded non-crystallizable component in the amorphous melt region remains unclear. Understanding the concentration of the excluded non-crystallizable component in the amorphous melt region is significant for providing insights into the miscible state and crystallization mechanisms of polymer blends.

The aim of this present study is to gain a deeper understanding of the concentration of the non-crystallizable component in the excluded non-crystallizable amorphous region. To this end, we investigated the spherulite growth of crystalline/crystalline polymer blends of poly(trimethylene terephthalate) (PTT) and poly(ethylene terephthalate) (PET), which are miscible at temperatures above about 210 °C [37]. The difference in the melting temperature *T*_m_ of PET and PTT (258 and 238 °C, respectively) allows for the crystallization of PET and PTT separately; i.e., only PET crystallizes at the temperature above the *T*_m_ of PTT, PTT is excluded from the spherulite growth front of PET, and PTT crystallizes in the excluded PTT amorphous region with a decrease in temperature. The concentration of PTT is discussed in the context of a kinetic analysis of the spherulite growth of PTT in the excluded PTT amorphous region. The results of the small-angle X-ray scattering (SAXS) measurement ae also presented to discuss the exclusion of PTT from the interlamellar amorphous region. 

## 2. Materials and Methods

### 2.1. Materials 

PTT with an intrinsic viscosity (IV) of 1.7 dL/g and a melting temperature *T*_m_ of 228 °C was provided by Asahi Chemical, Inc. (Tokyo, Japan). PET with an intrinsic viscosity (IV) of 0.45 dL/g and *T*_m_ of 258 °C was provided by Teijin, Inc. (Tokyo, Japan). 

### 2.2. Preparation of PTT/PET Blends

PTT and PET were melt-mixed in a mixing chamber (Imoto IMC-18D7, Kyoto, Japan) at a temperature of 300 °C and a rotor speed of 200 rpm for a period of 5 min. Prior to the melt blending process, the PTT and PET were subjected to a drying process in a vacuum oven at 100 °C for a period of 24 h. This process was performed to prevent transesterification during the melt blending process. The blend specimens were compression-molded between two metal plates at 300 °C for 1 min and then quenched in ice water to obtain a film specimen of approximately 100 μm thickness. 

### 2.3. Characterization

#### 2.3.1. Polarized Optical Microscopic Observation 

The film sample was melted at 300 °C for 1 min on a hot stage and then immediately transferred to another hot stage on the stage of the polarized optical microscope and annealed at the desired crystallization temperature for melt crystallization. A polarized optical microscope (Olympus BX53, Tokyo, Japan) equipped with a sensitive tint plate, with an optical path difference of 530 nm and a charge-coupled device (CCD) camera (Olympus DP74, Tokyo, Japan), was used to observe the spherulite growth behavior during the melt crystallization. The morphology change of the melt-crystallized specimen during annealing after cooling and heating at a rate of 20 °C/min was also observed with the polarized optical microscope equipped with the hot stage. The morphology change observed by the CCD camera was recorded by optional software (Olympus cellSens Dimension 3.2, Tokyo, Japan). The spherulite radius at different crystallization times was obtained with an error of 0.2 μm using the measurement function of the Olympus cellSens Dimension 3.2 software. To check the reproducibility, spherulites with different distances between the neighboring spherulites were selected among the spherulites observed in the recorded area of 357 μm in length and 223 μm in width, and the spherulite radius versus time was obtained for more than three spherulites at each different temperature and blend content.

#### 2.3.2. Small-Angle X-ray Scattering Measurements

The small-angle x-ray scattering (SAXS) experiment for the melt-crystallized specimen was performed using the NANO-Viewer system (Rigaku Co., Ltd., Tokyo, Japan). For the SAXS experiment, the structure of the melt-crystallized specimen was frozen by quenching in ice water after the melt crystallization. Cu-Ka radiation with a wavelength of 0.154 nm was generated at 46 kV and 60 mA and was collimated by a confocal max-flux mirror system. Measurements were performed at room temperature, and the exposure time was 1 h. An imaging plate (IP) (BAS-SR 127, Fujifilm Corp., Tokyo, Japan) was used as a two-dimensional detector to obtain scattering images. The scattering images thus obtained were converted into text data by an IP reader (RAXIA-Di, Rigaku Co., Ltd., Tokyo, Japan). The scattering intensities were corrected for exposure time, sample thickness, and transmittance.

## 3. Results and Discussions

### 3.1. Exclusion of PTT during the Spherulite Growth of PET 

**Figure 1:**
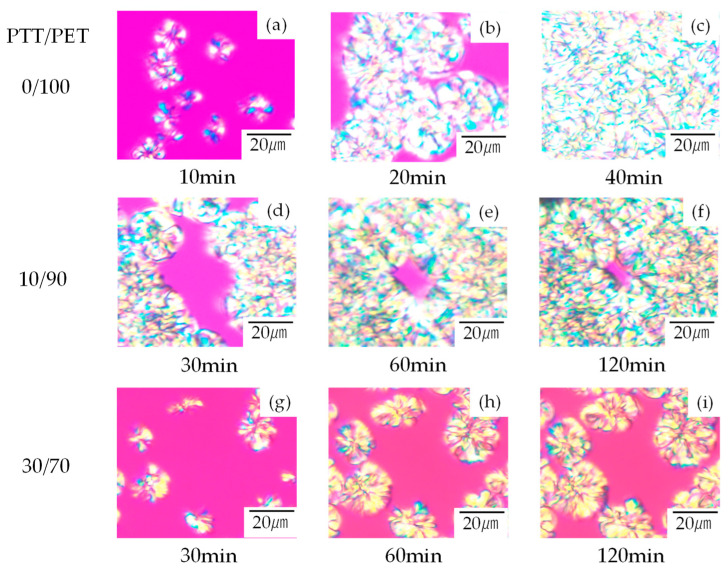
Structural evolution of PTT/PET blends with different PTT contents at various times during the melt crystallization at 240 °C observed by polarized optical microscope: (**a**–**c**) neat PET, (**d**–**f**) 10/90 PTT/PET, (**g**–**i**) 30/70 PTT/PET.

Figure 1 shows the evolution of spherulite growth in miscible crystalline/crystalline blends of PTT/PET with different PTT contents during the melt crystallization process at 240 °C. Since the crystallization temperature of 240 °C was below the melting temperature *T*_m_ of PET (258 °C) and was above that of PTT (238 °C), PTT did not crystallize, but only PET crystallized. The spherulites increased in size with time, and the space was eventually filled with spherulites in the neat PET (0/100 PTT/PET) (Figure 1a–c). Of particular interest is the fact that the growth of the PET spherulites stopped in the blends before the volume filling, leaving a non-crystallized amorphous region (Figure 1d–i). The non-crystallized amorphous region became larger as the PTT content increased (Figure 1f,i). Since the PTT and PET were miscible at 240°C as reported in our previous work [37], the non-crystallized amorphous region is not due to the segregated PTT by liquid–liquid phase separation, but to the exclusion of the PTT from the growth front of the PET spherulites. No significant change in spherulite morphology was observed in the blends, suggesting exclusion into the interspherulite amorphous region. This is different from the change to coarse and open spherulites due to exclusion into the interfibrillar amorphous region, which is usually observed in polymer blends [15,35]. Inhibition of the crystallization by PTT prevented the nucleation and growth of PET spherulite at 240 °C when the PTT content was above 30 wt%. 

**Figure 2:**
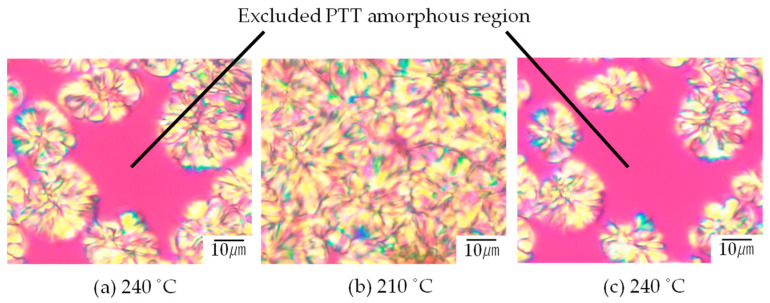
Morphology change of 30/70 PTT/PET by cooling and heating: (**a**) melt crystallization at 240 °C, (**b**) cooling to 210 °C, (**c**) heating to 240 °C.

Figure 2 shows the morphology change with cooling and heating for the PET spherulite and the non-crystallized amorphous region in 30/70 PTT/PET obtained by melt crystallization at 240 °C shown in Figure 1i. As a result of the temperature being lowered from 240 °C to 210 °C and annealing, the non-crystallized amorphous region shown in Figure 1i was volume-filled with spherulites (Figure 2a,b). This indicates that the crystallization was induced and volume-filled when the temperature was lowered to the temperature below the *T*_m_ of PTT (238 °C). When the temperature was raised to 240 °C, which is above the *T*_m_ of PTT and below that of PET, the spherulite obtained at 210 °C disappeared, but the PET spherulite obtained by crystallization at 240 °C shown in Figure 2a remained (Figure 2c). These results confirm that the non-crystallized amorphous region observed in the blends shown in Figure 2a (Figure 1i) can be attributed to the excluded PTT, which was obtained by excluding PTT from the spherulite growth front of PET through the melt crystallization process at 240 °C.

### 3.2. Three-Stage Spherulite Growth Process of PET by Exclusion of PTT

**Figure 3:**
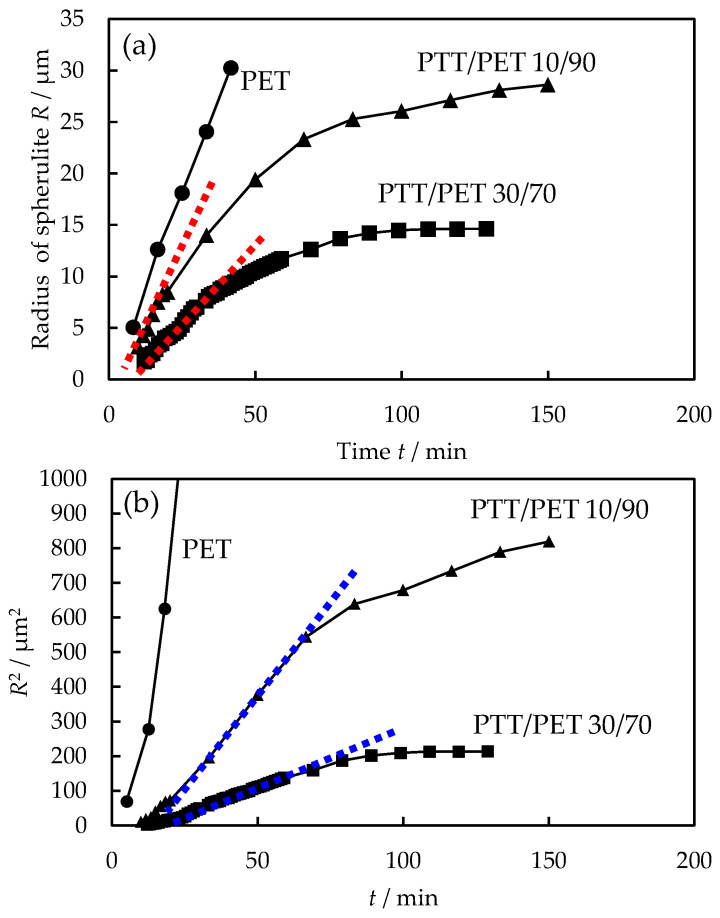
Time *t* variation of spherulite radius *R* during the melt crystallization of PTT/PET blends with different PTT contents at 240 °C: (**a**) *R* vs. *t*, (**b**) *R*^2^ vs. *t*.

Figure 3 shows the representative data for the time t variation of the spherulite radius *R* during the melt crystallization of PTT/PET blends with different PTT contents at 240 °C, which was obtained from the growth of the PET spherulites shown in Figure 1. The radius *R* increased linearly with time t in the neat PET, *R* ∝ *t*, indicating a constant spherulite growth rate *G* = d*R*/d*t*, *G* ∝ *t*^0^. The spherulite growth rate slowed in the blends, and it decreased with increasing the PTT content (Figure 3a). The *R* in the blends also showed an initial linear increase with *t*, as indicated by the dashed red line. However, after the initial linear increase, the *R* exhibited a nonlinear increase with t. This indicates a decrease in the spherulite growth rate *G* with time *t*, as has been observed in a number of different polymer blends [4,7,8,9,12,14,15]. 

After the initial linear increase in radius *R* with time *t*, the *R*^2^ showed a linear increase with *t* in the blends, as indicated by the dashed blue line (Figure 3b). The linear increase in *R*^2^ with *t* indicates *R* ∝ *t*^1/2^ and *G* ∝ *t*^−1/2^. The spherulite growth of *G* ∝ *t*^−1/2^ can be explained by a diffusion-controlled crystallization process occurring under a concentration gradient [8,36,38]. Thus, the spherulite growth rate changed from *G* ∝ *t*^0^ in the initial stage (stage I) to *G* ∝ *t*^−1/2^ in the subsequent stage (stage II). The change in the *G* from *G* ∝ *t*^0^ to *G* ∝ *t*^−1/2^ has been proposed as a possible mechanism in a number of other blend systems such as polypropylene/liquid paraffin [8], poly(butylene terephthalate)/poly(ε-caprolactone) [12], poly(butylene succinate)/poly(butylene adipate) [14], isotactic polypropylene/partially hydrogenated oligo(styrene-co-indene) [11], poly(ethylene oxide)/ethylene-methacrylic acid [32], poly(ethylene oxide/styrene-hydroxy styrene [32], and isotactic polystyrene/polyphenylene oxide [39]. The increase in *R* did not follow the relationship of *R* ∝ *t*^1/2^ at the longer time due to a further decrease in the spherulite growth rate, which was attributed to the slow spherulite growth process (stage III). 

**Figure 4:**
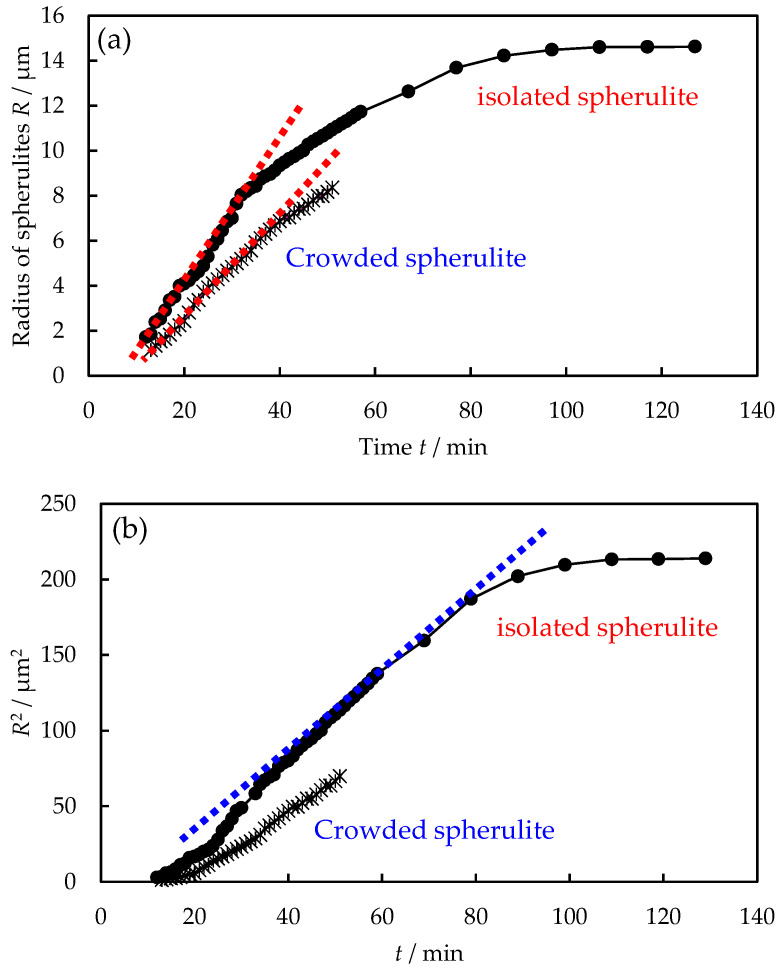
Time *t* variation of spherulite radius *R* during the melt crystallization of 30/70 PTT/PET at 240 °C for different distances between the neighboring spherulites: (**a**) *R* vs. *t*, (**b**) *R*^2^ vs. *t*.

As shown in Figure 4, the spherulite growth behavior of the blends showed significant differences with different distances between the neighboring spherulites due to the difference in the PTT concentration in the PTT excluded amorphous region. The three spherulite growth stages of the blends proposed in Figure 3 were observed in the isolated spherulite in which the distance between the neighboring spherulites is long, over 70 μm, i.e., *R* ∝ *t* in the initial stage (Figure 4a), and it changed to *R* ∝ *t*^1/2^ in the subsequent stage (Figure 4b). In contrast, in the crowded spherulite, where the distance between the neighboring spherulites is short, below 50 μm, the spherulite growth did not follow *R* ∝ *t*^1/2^ in the later stage (Figure 4b), while it followed *R* ∝ *t* in the initial stage (Figure 4a). The deviation from the relationship of *R* ∝ *t*^1/2^ in the crowded spherulites is attributed to the interference of the excluded PTT in the amorphous region between the neighboring spherulites due to the continuous exclusion of PTT from the spherulite growth front of the neighboring spherulites, as observed in polypropylene/liquid paraffin blends [8]. 

**Figure 5:**
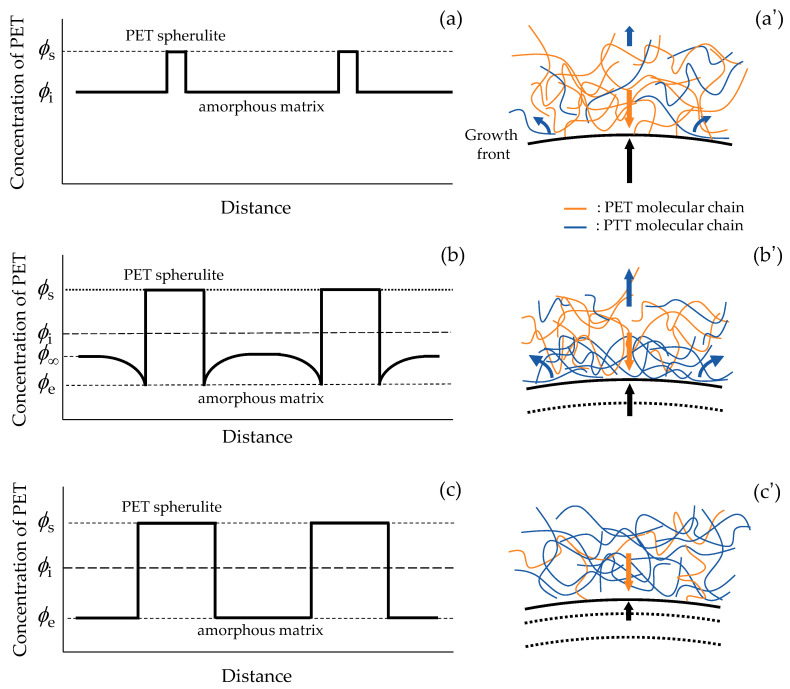
Schematic illustration of the change in concentration profile and exclusion from the spherulite growth front at different stages during spherulite growth of PET in 30/70 PTT/PET blends: (**a**,**a’**) stage I, (**b**,**b’**) stage II, (**c**,**c’**) stage III. Black arrow: spherulite growth, orange arrow: deposition of PET chain on the growth front, blue arrow: exclusion of PTT chain from the growth front.

Figure 5 shows a schematic illustration of the three spherulite growth stages of PTT/PET blends proposed in Figure 3. Here, the PET concentration in the initial amorphous melt state and that of the PET spherulite are assumed to be *ϕ*_i_ and *ϕ*_s_, respectively. In stage I, the PET spherulite nucleates and grows from the initial amorphous melt state, and the non-crystallizable PTT component is inserted into the interlamellar amorphous region. Since the exclusion of PTT is confined to the interlamellar amorphous region, and the PET concentration at the spherulite growth front is equal to that of the initial amorphous melt state *ϕ*_i_ during the spherulite growth (Figure 5a,a’), the spherulite grows by *G* ∝ *t*^0^ (*R* ∝ *t*^1^). This is because there is no significant change in the PTT concentration at the growth front. In the miscible blends, the spherulite growth is controlled by the diffusion of the crystallizable PET chain and is deposited on the spherulite growth front as indicated by an orange arrow and by that of the exclusion of non-crystallizable PTT chain from the growth front as indicated by a blue arrow (Figure 5a’). This competitive situation is called mutual diffusion [40]. In stage II, the spherulite growth rate decreases with time, and the spherulite grows by *G* ∝ *t*^−1/2^ (*R* ∝ *t*^1/2^) due to the increase in the PTT concentration at the growth front caused by the exclusion of PTT from the spherulite growth front. The characteristic spherulite growth of *G* ∝ *t*^−1/2^ (*R* ∝ *t*^1/2^) in stage II suggests that the crystallization kinetics are governed by a diffusion-controlled crystallization process and a concentration gradient is formed at the growth front of the PET spherulite (Figure 5b,b’). According to Cahn’s kinetic theory, the growth of the PET spherulite domain, which is subject to a diffusion-controlled process, is given by the following equation corresponding to *R* ∝ *t*^1/2^ [8,36,38]:

(1) $$R^{2}=[(\phi_{\infty }-\phi_{e})D/\phi_{S}]t$$

where *ϕ*_∞_ is the PET concentration at a point far from the growth front in the amorphous region, *ϕ*_e_ is the equilibrium concentration at the growth front of the PET spherulite, *ϕ*_s_ is the PET concentration of the PET spherulite, and *D* is the diffusion coefficient. Subsequently, the concentration distribution decreases in the amorphous region and then disappears in the later stage III, resulting in a close proximity of *ϕ*_∞_ to *ϕ*_e_, and the spherulite grows by deposition of the crystallizable PET from the high-PTT-content excluded PTT amorphous region (Figure 5c,c’). Due to the absence of a diffusion-controlled crystallization process by a concentration distribution and the decrease in PET concentration (*ϕ*_∞_ and *ϕ*_e_) during the spherulite growth, the spherulite growth rate in stage III is significantly slower than that observed in stage II.

### 3.3. Spherulite Growth in the Excluded PTT Amorphous Region

**Figure 6:**
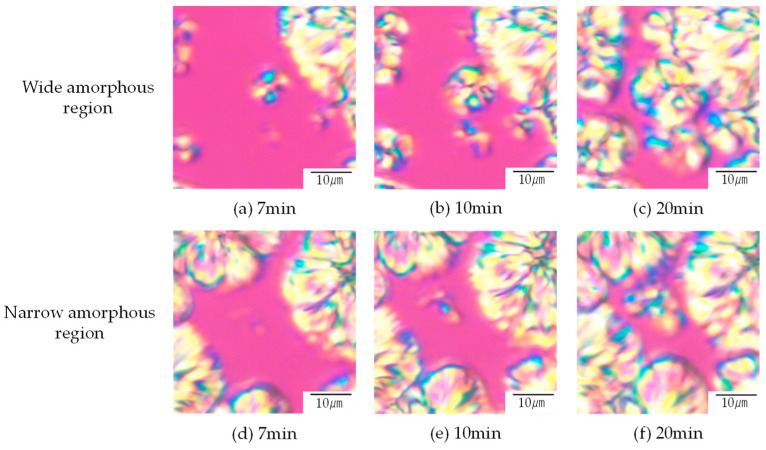
Spherulite growth of PTT in the excluded PTT amorphous region in 30/70 PTT/PET at 210 °C after melt crystallization at 240 °C: (**a**–**c**): wide excluded amorphous region between isolated spherulites, (**d**–**f**): narrow excluded amorphous region between crowded spherulites.

As described above, the existence of the excluded PTT amorphous region was suggested. To clarify the excluded PTT amorphous region, Figure 6 shows the spherulite growth of the PTT in the excluded PTT amorphous region in 30/70 PTT/PET at 210 °C after melt crystallization at 240 °C. The PTT spherulite grew in the excluded PTT amorphous region. The number density of the PTT spherulites in the wide amorphous region was higher, and the nucleation of the PTT spherulites started faster than that observed in the narrow amorphous region. In the wide amorphous region formed by isolated spherulites, the PTT spherulites grew randomly around the pre-existing PET spherulites as well as in the central region (Figure 6a–c). In contrast, in the narrow amorphous region formed by crowded spherulites, the PTT spherulites grew only around the central region (Figure 6d–f). This difference can be attributed to the different PTT concentrations in the narrow and wide excluded PTT amorphous regions formed by the isolated and crowded spherulites, respectively.

**Figure 7:**
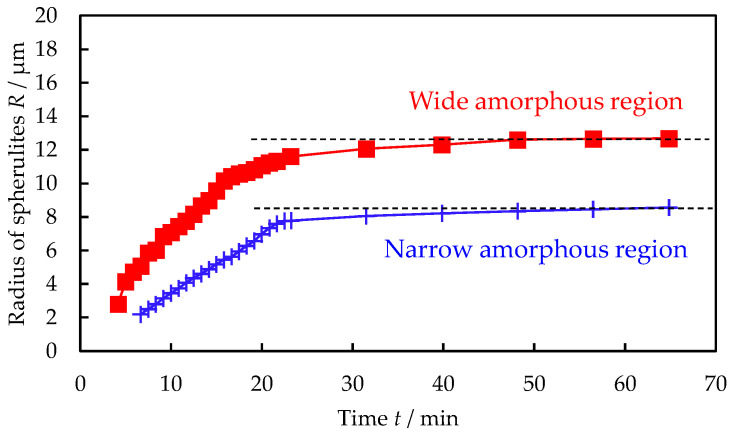
Time *t* variation of spherulite radius *R* for different areas during the melt crystallization of PTT in excluded PTT amorphous region in 30/70 PTT/PET at 210 °C after melt crystallization at 240 °C.

Figure 7 shows the time *t* variation of the spherulite radius *R* during the melt crystallization of PTT in the excluded PTT amorphous region in 30/70 PTT/PET at 210 °C, which was obtained from the spherulite growth shown in Figure 6. The *R* increased nonlinearly with *t* during the crystallization of PTT; i.e., the increase in *R* with *t* became smaller due to the decrease in the spherulite growth rate with *t*. The nonlinear spherulite growth rate can be attributed to the existence of PET in the excluded PTT amorphous region and the exclusion of PET during the crystallization of PTT. The initial growth rate, estimated from the initial slope of *t* vs. *R* in the wide amorphous region, was observed to be faster than that in the narrow amorphous region. This suggests that the PTT concentration in the wide amorphous region was lower than that in the narrow amorphous region.

**Figure 8:**
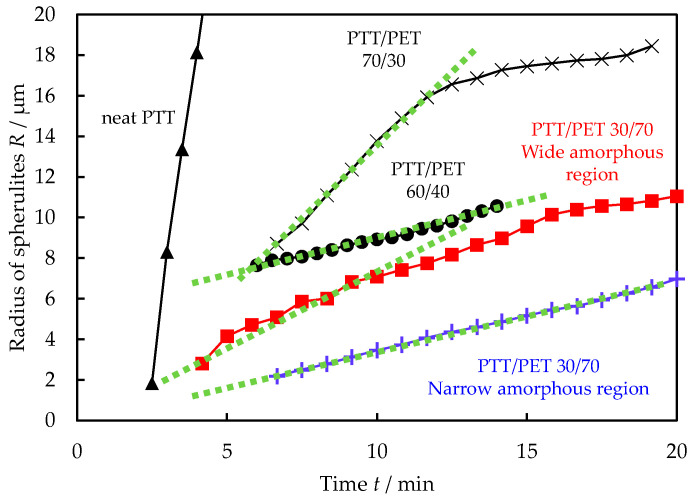
Time *t* variation of spherulite radius *R* during the melt crystallization of PTT in 30/70 PTT/PET at 210 °C after melt crystallization at 240 °C, and those of the melt crystallization of the neat PTT and the high-PTT-content PTT/PET blends at 210 °C.

To estimate the PTT concentration in the excluded PTT amorphous region in 30/70 PTT/PET, the time *t* variations of the spherulite radius *R* given in Figure 7 are shown in Figure 8 for comparison with those of the neat PTT and the PTT/PET blends with high PTT content during the crystallization at 210 °C. To avoid any confusion, the spherulite growth at the late stage is shown for the high-PTT-content blends, since the PTT spherulites grew only in the late stage after the PET spherulites grew in the early stage. The spherulite growth rate, estimated by the initial slope of the *t* vs. *R*, in the excluded PTT amorphous region was found to be significantly slower than that of the neat PTT, indicating that the excluded PTT amorphous region is not composed of PTT alone, but also contains PET. The growth rate of the crystallization process in the excluded PTT amorphous region was found to be intermediate between that of the 60/40 and 70/30 PTT/PET blends. These results suggest that the PTT concentration in the range of 60 to 70% is contained in the excluded PTT amorphous region. This implies that the PTT concentration in the amorphous region changes from the initial PTT content of 30% to 60–70% by melt crystallization at 240 °C as a result of the exclusion of PTT from the growth front of PET spherulites.

### 3.4. Evidence for Exclusion of PTT from the Interlamellar Region

**Figure 9:**
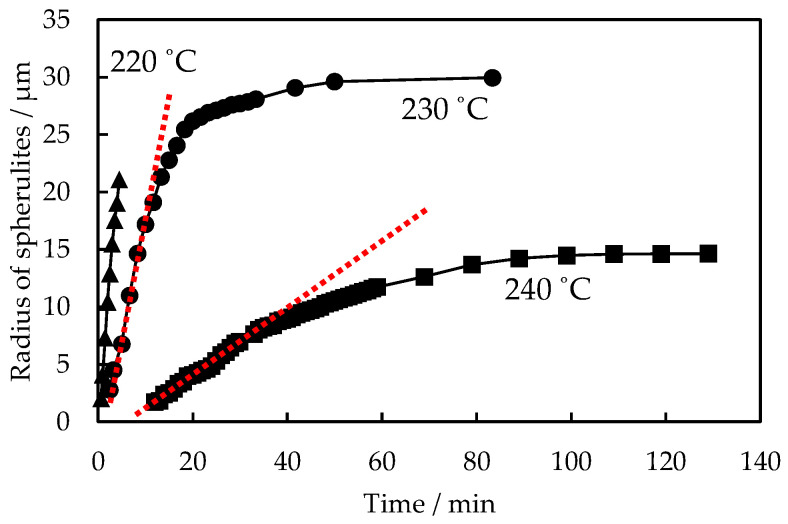
Time *t* variation of spherulite radius *R* during melt crystallization of 30/70 PTT/PET at various temperatures.

Figure 9 shows the time *t* variation of the spherulite radius *R* during the melt crystallization of 30/70 PTT/PET at different crystallization temperatures between 220 °C and 240 °C. As the crystallization temperature increased, the spherulite growth rate *G* decreased, and the linear spherulite growth did not continue to a larger spherulite size, but the nonlinear spherulite growth started at a smaller spherulite size. Therefore, the nonlinear spherulite growth region became more obvious as the crystallization temperature increased. The difference can be attributed to the different amounts of PTT excluded from the growth front of the PET spherulites with different crystallization temperatures. The exclusion of PTT can be quantitatively explained by a parameter *δ* proposed by Keith and Padden [29,41]:

(2) $$\delta =\frac{D}{G}$$

where *δ* is the distance at which the non-crystallizable component (PTT) can be excluded from the crystal growth front, *D* is the diffusion coefficient of the non-crystallizable component, and *G* is the growth rate of the crystallizable component (PET). As the crystallization temperature increases, *D* increases while *G* decreases, resulting in an increase in the value of the parameter *δ*. This indicates that the amount of excluded PTT increases and the nonlinear spherulite growth rate region becomes more obvious as the crystallization temperature increases.

**Figure 10:**
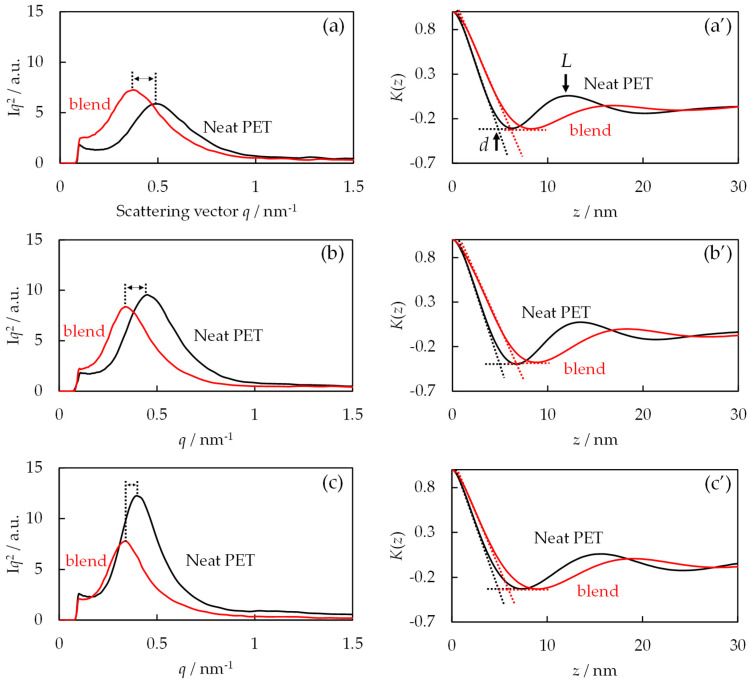
Lorentz-corrected SAXS intensity profiles and one-dimensional correlation function *K*(*z*) of the neat PET and 30/70 PTT/PET blends obtained by melt crystallization at various temperatures: (**a**,**a’**) 220 °C, (**b**,**b’**) 230 °C, (**c**,**c’**) 240 °C. *d*: lamellar thickness, *L*: long period.

Figure 10a–c shows the Lorentz-corrected SAXS intensity profiles of the neat PET and 30/70 PTT/PET blends obtained by melt crystallization at different crystallization temperatures *T*c ranging from 220 °C to 240 °C. Here, the blends were crystallized until the spherulite growth of the PET stopped and then quenched in ice water to prevent further crystallization in the PTT excluded amorphous region, and the scattering intensity *I* was multiplied by the square of the scattering vector *q* for Lorentz correction. A single peak was observed at the peak position of the scattering vector *q*_max_ in both the neat PET and the blends, indicating the regular arrangement of lamellae in the stacked lamellae. The *q*_max_ of the blends was observed at a lower value than that of the neat PET, and the difference was smaller with increasing *T*c, indicating a difference in the structure of the stacked lamellae depending on *T*c.

In order to estimate the detail of the structure in the stacked lamellae to understand the exclusion from the interlamellar amorphous region, the thickness of the interlamellar amorphous region *AM* was obtained by the one-dimensional correlation function *K*(*z*), which was calculated by Fourier transformation of the SAXS intensity profiles in Figure 10a–c using the following equation [18,30]:

(3) $$K\left(z\right)=\frac{1}{2\pi }\int_{0}^{\infty }q^{2}I\left(q\right)\cos qz\;dq$$

where *z* is the distance, *q* is the scattering vector, *I* is the scattering intensity. The correlation function *K*(*z*) thus obtained is shown in Figure 10a’–c’. The position of the first maximum indicates the long period *L*, which means the most probable next neighboring distance of the lamellae. The average lamellar thickness in the stacked lamellae *d* can be obtained from the intersection of the extrapolation of the initial slope for *K*(*z*) and the horizontal line for the first minimum of the *K*(*z*) [18,30]. The thickness of the interlamellar amorphous region *AM* was obtained by *AM* = *L* − *d*. The *L*, *d*, *AM* of the neat PET and 30/70 PTT/PET blends crystallized at different temperatures *T*c thus obtained are shown in Table 1. The *AM* of the blend *AM*_blend_ was larger than that of the neat PET *AM*_PET_, indicating the presence of PTT in the interlamellar amorphous region of the PET spherulites. The difference in *AM*, Δ*AM* = *AM*_blend_ − *AM*_PET_, at the crystallization temperature *T*c of 220 °C, 230 °C, and 240 °C was 3.4 nm, 3.2 nm, and 2.1 nm, respectively. Thus, the Δ*AM* decreased with an increase in the crystallization temperature, which is associated with an increase in the amount of excluded PTT with increasing temperature, as expected from Equation (2). Due to the increase in the amount of PTT excluded from the spherulite growth front of PET with increasing crystallization temperature, the nonlinearity region in the time variation of the spherulite radius became more obvious, as shown in Figure 9. These results support the hypothesis that the characteristic spherulite growth process observed in the 30/70 PTT/PET blend by melt crystallization at 240 °C (as shown in Figure 1, Figure 3 and Figure 4) can be attributed to the significant exclusion of PTT from the interlamellar amorphous region into the interspherulite amorphous region.

## 4. Conclusions

In the miscible crystalline/crystalline blends of PTT/PET, the PET spherulites grew at a nonlinear rate, i.e., *G* ∝ *t*^0^ (*R* ∝ *t*^1^) in stage I, *G* ∝ *t*^−1/2^ (*R* ∝ *t*^1/2^) in stage II, and relatively slow growth in stage III, due to the exclusion of PTT from the spherulite growth front of PET by melt crystallization at 240 °C. Due to the exclusion, the PET spherulites stopped growing before volume filling, causing the formation of an excluded PTT amorphous region. When the temperature was lowered from 240 °C to 210 °C, the PTT spherulites grew in the excluded PTT amorphous region. The growth rate of PTT spherulites in the excluded PTT amorphous region was not equivalent to that of the pure PTT, but rather to that of the blends of 60–70 wt% PTT concentration, indicating that the PTT concentration in the excluded PTT amorphous region is 60–70 wt%. These results suggest that the PTT concentration in the amorphous region changes from the initial PTT content of 30 wt% to 60–70 wt% due to the exclusion from the spherulite growth front of PET during the spherulite growth of the PET. The exclusion of PTT from the interlamellar amorphous region was confirmed by the SAXS results. Understanding the concentration of the non-crystallizable component in the excluded non-crystallizable amorphous region and the resulting characteristic crystallization behavior provides promising insights into the miscible state and crystallization mechanisms of polymer blends, which is important for controlling the crystalline morphology and the resulting properties. As discussed in Figure 5, the concentration distribution of the non-crystallizable component is also changed in the excluded non-crystallizable region during the spherulite growth. To quantitatively estimate the concentration distribution, scanning transmission electron microscopy (STEM)–energy-dispersive X-ray spectroscopy (EDS) elemental analysis [42] could be a promising method, although this method is limited to the blends used.

## Figures and Tables

**Table 1:** Long period *L*, lamellar thickness *d*, thickness of interlamellar amorphous region *AM* of the neat PET and 30/70 PTT/PET crystallized at different temperatures obtained from Figure 10a’–c’.

*T*c/°C	PET				Blend			Δ*AM*/nm
	*L*/nm	*d*/nm	*AM*/nm		*L*/nm	*d*/nm	*AM*/nm	
220	12.2	4.8	7.4		16.9	6.1	10.8	3.4
230	13.4	5.1	8.3		18.2	6.7	11.5	3.2
240	15.7	5.1	10.6		18.9	6.2	12.7	2.1

## Data Availability

The original contributions presented in the study are included in the article, further inquiries can be directed to the corresponding author.
